# Semalytics: a semantic analytics platform for the exploration of distributed and heterogeneous cancer data in translational research

**DOI:** 10.1093/database/baz080

**Published:** 2019-07-09

**Authors:** Andrea Mignone, Alberto Grand, Alessandro Fiori, Enzo Medico, Andrea Bertotti

**Affiliations:** 1 Candiolo Cancer Institute, FPO, IRCCS, Candiolo, Torino, Italy; 2 Department of Oncology, University of Torino, Torino, Italy

## Abstract

Each cancer is a complex system with unique molecular features determining its dynamics, such as its prognosis and response to therapies. Understanding the role of these biological traits is fundamental in order to personalize cancer clinical care according to the characteristics of each patient’s disease. To achieve this, translational researchers propagate patients’ samples through *in vivo* and *in vitro* cultures to test different therapies on the same tumor and to compare their outcomes with the molecular profile of the disease. This in turn generates information that can be subsequently translated into the development of predictive biomarkers for clinical use. These large-scale experiments generate huge collections of hierarchical data (i.e. experimental trees) with relative annotations that are extremely difficult to analyze. To address such issues in data analyses, we came up with the Semalytics data framework, the core of an analytical platform that processes experimental information through Semantic Web technologies. Semalytics allows (i) the efficient exploration of experimental trees with irregular structures together with their annotations. Moreover, (ii) the platform links its data to a wider open knowledge base (i.e. Wikidata) to add an extended knowledge layer without the need to manage and curate those data locally. Altogether, Semalytics provides augmented perspectives on experimental data, allowing the generation of new hypotheses, which were not anticipated by the user *a priori*.

In this work, we present the data core we created for Semalytics, focusing on its semantic nucleus and on how it exploits semantic reasoning and data integration to tackle issues of this kind of analyses. Finally, we describe a proof-of-concept study based on the examination of several dozen cases of metastatic colorectal cancer in order to illustrate how Semalytics can help researchers generate hypotheses about the role of genes alterations in causing resistance or sensitivity of cancer cells to specific drugs.

## Introduction

Understanding cancer dynamics is essential for biomedical research. Studies of past decades have shown that tumors are not identical instances of a universal disease prototype (e.g. breast cancer and lung cancer). Although they affect the same tissues and they often share very similar phenotypes, it has been demonstrated that each cancer is a complex and variable system with unique characteristics at the molecular level. This is ascribable to the Darwinian selection of stochastic events (i.e. genetic alterations), which is inherently responsible for cancer onset and progression (1). Those causative events determine the intrinsic genomic features that, in turn, are responsible for cancer behavior and therefore may be exploited to predict the effectiveness of candidate therapies or the prognosis of the disease. Therefore, grasping and leveraging correlations between genome variants and drug responses are fundamental to optimize personalized clinical care in precision medicine (2).

Understanding how genomic alterations affect drug responses is the main goal of translational research in cancer pharmacogenomics, where pre-clinical investigators work close to the clinic in order to move relevant discoveries from bench to bedside. In this context, patient-derived tumor specimens are (i) exploited to generate cultures, which are then serially propagated through *in vivo* or *in vitro* experimental procedures (e.g. propagation in mice, cell cultures) to create distinct biological samples (i.e. bioentities) derived from the same tumor (3, 4) and/or (ii) transformed to extract information about the molecular characteristics of the tumor. Usually, the final goal of such approaches is to match the spectrum of efficacy of different drugs against the molecular configuration of a specific cancer case.

The collection of bioentities (e.g. cell lines, tissues, mice and DNA/RNA aliquots), together with their related data, is likely to shape a hierarchical data structure, in which the biospecimens are nodes interconnected by links representing the experimental procedures through which they have been generated or transformed. We call such hierarchies ‘experimental trees’. Experimental trees are characterized by a variable degree of complexity, which is determined by multiple factors.

1. **Heterogeneous observations:** different kinds of bioentity are exploited to generate dissimilar observations types (i.e. experimental results). For instance, treated mice can produce observations about therapies performance, instead derived DNA aliquots can create molecular observations.
2. **Longitudinal series**: the repetition of the same measures in time. For example, administered therapies could alter genomic features of cancer in different ways, which, in turn, can modify its downstream behavior (e.g. resistance to therapies and prognosis; 5). Monitoring those changes requires one to collect chronological sequences of measures sets.
3. **Bioentities branching**: the isolation of longitudinal experiments in distinct branches to test diverse treatment strategies and to simulate reactions and modifications of the same cancer under different conditions.

For instance, the DNA of a tumor can be extracted from a sample and sequenced. Then, a different biological specimen, derived from the same sample, can be implanted subcutis in an immunocompromised mouse to test *in vivo* the efficacy of a specific drug. Finally, at the end of the therapy, the tumor can be retrieved from the mouse for a new DNA extraction and a new sequencing; besides it can be used to generate new bioentities downstream. In this way, each sample produces heterogeneous data (e.g. genomic profiles and drug sensitivity data) obtained through experiments performed at different times on different nodes of the experimental branch (e.g. before and after the treatment).

This hierarchical representation of experimental activities enables the systematic comparison of genomic landscapes before and after therapies, which is instrumental for the identification of recurring gains or losses of specific genetic variants occurring during treatments. Therefore, for analytical purposes, heterogeneous data scattered along experimental trees of variable size have to be summarized and compared according to the investigational needs.

**Figure 1:**
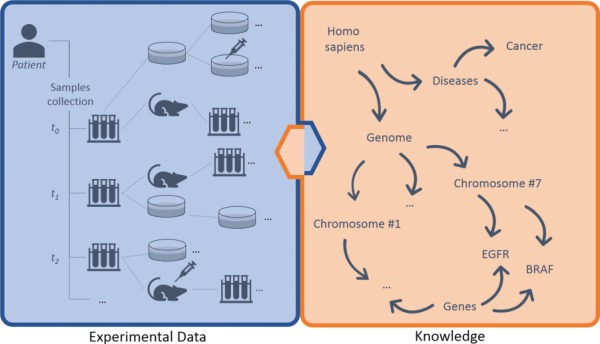
Large-scale experiments in translational research. On the left, the production of operational data derived from patient’s samples, which generate hierarchical structures with several bioentities. On the right, the knowledge that describes abstract concepts. Each starting sample can be serially propagated in experimental branches to assess clinical hypotheses (e.g. the sensitivity of cancer cells to specific drugs) and to get insights about ‘-omics’ data. Results should be annotated along branches by virtually connecting experimental reports, scattered along nodes, to biological features in knowledge. New results create new knowledge, strengthening the loop of translational cancer research.

Breaking down data types involved, we can distinguish three main layers of information: (i) experimental data, (ii) knowledge and (iii) annotations (Figure 1). The (i) experimental data describe streams of bioentities in trees and their connections. The (ii) knowledge represents the abstract set of interconnected metadata that defines general concepts and rules involved in pharmacogenomics (e.g. *BRAF* is a human gene; it encodes a protein called B-Raf). The (iii) annotation data type connects experimental data to the knowledge to keep track of observations linked to bioentities along trees. The management, the curation and the analysis of this three-tier information space is crucial for the success of these translational approaches.

Data that characterize modern science are constantly increasing. Both experimental information and knowledge have been rapidly accumulating at exponential rates. Indeed, the capabilities of high-throughput methods have made laboratory data more affordable and easier to produce. For example, next-generation sequencing technologies have dramatically reduced costs associated with genome sequencing and, more in general, major improvements in biotechnologies have eased the extraction of genomic information (6). This avalanche of operational data generates a similar trend also in the rate of knowledge accumulation (7). Newly generated knowledge sets the bases for the production of new hypotheses. As a consequence, new experimental data are produced, enlarging analytics perspectives and further reinforcing this virtuous loop.

Such advancements enable large-scale translational research settings oriented to pharmacogenomics. Indeed, the creation of huge collections of experimental trees with large branches can be deployed at affordable costs in order to match numerous candidate treatments against genomic features. These practices frequently produce huge collections of trees composed by thousands of hierarchical nodes representing bioentities and their related data. From the data management perspective, this requires an information technology (IT) tool for helping researches get relevant information in large-scale hierarchical scenarios.

To the best of our knowledge, there are no IT platforms devoted to this specific aim, and these data are managed with *ad hoc* solutions or using common software. For example, tools like the Laboratory Information Management Systems (LIMSs) are exploited to handle laboratory samples and processes (8). These are oriented to daily data-tracking and are usually based on Online Transaction Processing Software (OLTP). In addition to LIMSs, analytical data portals, such as cBioPortal (9) and PDXFinder (10), focus on data analysis rather than on transactions processing and may rely on Online Analytical Processing Software (OLAP). However, LIMSs and data portals are not usually designed for dealing with annotations of hierarchical data. Moreover, they generally use controlled vocabularies (i.e. lists of metadata used as labels) as knowledge references for annotating biological information. This approach to data enrichment is affected by severe limitations. Indeed, on one hand it certainly allows querying experimental data tagged with a set of labels, for example a set of user-entered genes variants. However, on the other hand, it is almost impossible exploring relations among metadata in abstract knowledge. For example, one could be interested in querying samples annotated with all the sequence variants known to predict a positive response to a given drug. Usually, this preliminary knowledge items selection (i.e. the set of predictive variants to analyze) is performed manually, harnessing personal expertise, with no, or limited, help of machines. Of course, knowledge data require heavy and continuous curation to avoid getting outdated and to add new information. Such problems affect data processing, analysis and integration (11, 12).

In order to overcome such limitations in the data management in the presented translational settings, the following open issues need to be tackled.

1. **Tracking of experimental trees.** Bioentities and their relationships need to be tracked and queried in order to explore nodes along experimental trees with arbitrary lengths and irregular branches.
2. **Building a representation of knowledge.** A structured data representation is necessary to organize knowledge and describe biological features and their relationships. This need is crucial, since it represents the way to allow algorithms to investigate knowledge the way humans do. Moreover, knowledge data need to be updated and integrated with new information.
3. **Connecting experimental data with knowledge (annotation).** Heterogeneous experimental data should be connected to knowledge (i.e. annotated). In particular, a model accounting for the intrinsic interconnectedness of operational data with abstract concepts is required.
4. **Leveraging IT for working at scale.** Huge data volumes and complex data schemata require the use of specific computer technologies for querying huge hierarchical datasets.

In this work, we illustrate the design and the implementation of the data core of ‘Semalytics’ (the name comes from the synthesis of ‘Semantic Analytics’), an IT annotation platform based on Semantic Web technologies (13, 14) that aims to tackle such open issues. In particular, Semalytics helps users explore data with two major features: (i) it allows tracking, exploring and summarizing data and annotations scattered along experimental trees according to knowledge and (ii) it provides a framework for knowledge expansion through a real-time connection to Wikidata, the crowdsourced semantic project of Wikimedia Foundation (15). Finally, as a proof-of-concept, we use Semalytics to analyze drug–gene interactions in metastatic colorectal cancer, exploiting the platform to explore several experimental trees stored locally and connecting them to Wikidata.

## Platform implementation

We based Semalytics on a NoSQL architecture (16). The main reason for this choice is that the data issues presented in the previous section of this manuscript cannot be easily addressed harnessing Relational Database Management Systems (RDBMSs). Indeed, although RDBMSs represent a robust solution for several common applications, they do not fit well this specific scenario. In particular, the trees exploration requires queries similar to the ones used in social networks analysis (e.g. friend of a friend). Harnessing RDBMSs, the bioentities table should be recursively joined to explore trees and branches; however this implies a massive memory usage that renders the queries inefficient or even infeasible on large data collections (17, 18). The same scalability issue is further amplified when operational data are joined to knowledge through annotations, and data need to be retrieved together. Furthermore, RDBMSs present many drawbacks when the schema changes recurrently (19) like it happens in this context, where the data schema evolves frequently in order to extend knowledge entities and relations.

We started the design of the platform from the analysis of current features of the Laboratory Assistant Suite (http://las.ircc.it/), also known as the LAS: a custom LIMS developed in the Candiolo Cancer Institute (http://www.ircc.it), which is oriented to data management for translational research in oncology (20–23). LAS exploits a multilayered data architecture (i.e. polyglot persistence) built on the top of a hybrid storage framework created on both relational (i.e. MySQL) and NoSQL (i.e. Neo4j and MongoDB) Database Management Systems. In particular, the Neo4j-based layer of the system tracks data as a directed graph (http://neo4j.com/). With the use of Neo4j, nodes and links are labeled (i.e. classified) and are detailed with further key-value properties. This is extremely useful for tracking experimental trees. The LAS represents each bioentity as a typed node, which can be linked to its ancestor and to downstream bioentities derived from it (e.g. starting node, ‘Bioentity#1’; link, ‘generates’; ending node, ‘Bioentity#2’. The Bioentity#1 is a viable sample implanted in the Bioentity#2, a mouse). An analogous graph representation is used to assert structured knowledge facts (e.g. starting node, ‘PTEN’; link, ‘is_part_of’; ending node, ‘human_chromosome_10’). We built in the LAS a starting knowledge base, integrating external sources, such as Ensembl (24) and COSMIC (25). This knowledge graph describes concepts and relations among chromosomes, genomic regions, genes, exons, proteins and several genetic variants.

**Figure 2:**
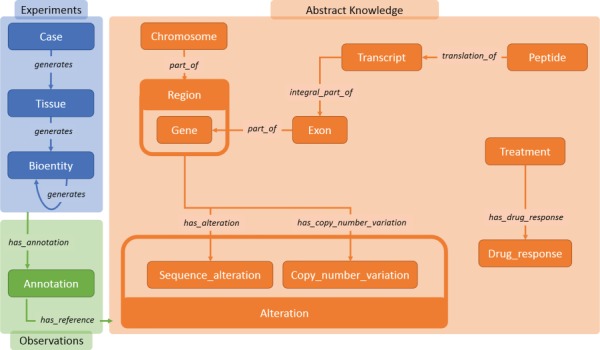
Main data classes in Semalytics. The blue area represents (i) operational data connected with the predicate ‘generates’. The orange region shows classes describing biological features in (ii) knowledge. The green zone illustrates the tracking of experimental observations, connecting operational information to abstract knowledge through (iii) annotation instances.

Although the adoption of Neo4j-based graph modeling enabled efficient hierarchical trees and graphs explorations, the current design of the LAS still suffers from requiring a massive amount of effort for data optimization and curation. Indeed, to improve the performance of specific traversal queries, it is sometimes essential building many additional links in the graph as a shorthand. In such cases, custom ‘CREATE’ queries or triggers are required, and/or *ad hoc* software is needed. Moreover, the knowledge is manually curated; therefore custom software is necessary in order to keep data up-to-date and to integrate new datasets. Indeed, the implementation of this local knowledge base has been requiring a lot of effort and time for manual integration of external datasets, and it requires continuous updates.

In order to overcome such restrictions, we created the data core of Semalytics modeling information with the Resource Description Framework (RDF), a standard data model created on purpose to describe and link information (26), which is at the basis of the Semantic Web technology (27). From the technical point of view, the Semantic Web is one of the most prominent ways for giving machines the capabilities to consume complex information, also in the context of scientific data curation (28). Its aim is building networks of Linked Data (i.e. a web of data), interconnected and extremely flexible, reflecting the structures of real world. The semantic ecosystem relies on the RDF that represents information as collections of triple patterns that describe graph-like structures. A triple consists of a subject, a predicate and an object. Resources (i.e. pieces of information) in triples are uniquely identified by Uniform Resource Identifiers (URIs), strings very similar to internet URLs, which can be used to identify data items and to make them likable ‘intra’*-* and ‘inter’*-*graph, borrowing the structure of the common Web. Additionally, Semantic Web is based on mathematical logic. Hence, given a set of inference rules, the starting graph can be extended through reasoning algorithms to infer new triples as a logical consequence of the starting set of assertions.

We built the data core of Semalytics relying on Semantic Web technologies. Harnessing this method, we can state triples about both experiments and knowledge with a standard data representation. Moreover, the graph structure and the logical reasoning allow efficient explorations of scattered data in trees and ease knowledge integration. For the practical implementation of Semalytics, we exploited GraphDB by Ontotext (https://www.ontotext.com/products/graphdb/), a triplestore (i.e. a database system for managing RDF graphs) that offers an infrastructure for dealing with Linked Data, bundled with a built-in reasoning engine. Furthermore, GraphDB has an integrated query endpoint based on SPARQL, a SQL-like declarative language for querying RDF data, available over HTTP. Semantic graphs can be founded on diverse standard logic rulesets: the more expressive is the logic, the deeper is the inference but the more expansive is its computational cost. In Semalytics, we chose OWL-Horst as inference ruleset, because of its scalability and since it represents a good balance between basic reasoning (i.e. mere syntactic reasoning) and more expressive and expansive rulesets (29).

**Figure 3:**
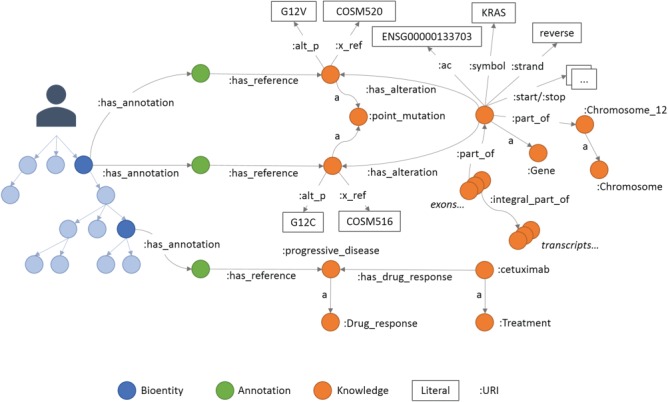
An example of annotations in Semalytics. On the left, colored with blue shadows, a small portion of a hierarchical tree where several bioentities are connected to annotation nodes (green dots in the middle) with predicates ‘has_annotation’. Annotation nodes are linked to knowledge items with predicates ‘has_reference’. On the right, a partial representation of knowledge with interconnected biological features. In this figure, the predicate ‘rdf:type’ is abbreviated ‘a’.

In the following sections, we denote with a colon the default prefix for local URIs defined in Semalytics (e.g. ‘:Gene’ indicates the Semalytics URI for the resource representing the category of human genes), and we use the Turtle serialization for the RDF to describe triples (30).

### Semalytics data model

The ontology (i.e. the description of entities and relations of a domain) of Semalytics defines general rules involved in (i) operational records, (ii) abstract knowledge and (iii) annotations. We exploited the RDF to define these data semantically (Figure 2).

We modeled (i) experimental data (blue area in Figure 2) with few categories: the class ‘Case’, which represents an anonymized reference to a patient with other metadata; ‘Tissue’, which characterizes the type of tissue collected; and ‘Bioentity’, which models the biological materials produced during experiments. Each of them can be subclassed by more specific categories for fine-grained typing (e.g. DNA, RNA, CellLine, etc.). All experimental data along trees belong to these classes. Indeed, since bioentities are propagated downstream through experimental procedures, every couple of instances can be connected through the predicate ‘generates’, which builds the main structure of tree branches. Harnessing this modeling philosophy, trees of arbitrary width and depth can be represented from a single root case.

Similarly, we included in Semalytics a structured representation of (ii) biological knowledge about human genome (orange area in Figure 2). The class ‘Chromosome’ collects all instances of human chromosomes. These instances are connected to objects belonging to the class ‘Region’, representing any range of nucleotides within a chromosome. A specific kind of region is the gene (i.e. a functional unit within the genome coding for molecules), which is described by the class ‘Gene’. Besides these general classes, we defined more specific physical and functional relations at the molecular level, for examples the classes ‘Exon’, categorizing the coding regions within genes; ‘Transcript’, representing the RNA instances derived from genes; and ‘Peptide’, which identifies the protein products of genes.

Furthermore, we also attempted to include part of the knowledge about the DNA alterations that are hallmarks of the oncological disease (31, 32). To do this, we introduced the main class ‘Alteration’, with several subclasses such as ‘Sequence_Alteration’ (e.g. point mutations) and ‘Copy_number_variation’ (e.g. amplifications).

Finally, we modeled the possible different outcomes of experiments in which tumors are subjected to therapies. The aim of this part of knowledge is to allow the categorization of treatments outcomes in response classes, depending on how tumor growth is affected by a therapy. In particular, the class ‘Treatment’ represents the type of therapeutic regimen, while the class ‘Drug_response’ indicates the class of the response to therapies. Each instance of the class ‘Drug_response’ is connected to its possible response types (e.g. see Figure 3). In order to classify the response, we use a clinical*-‘*like’ assessment, based on the tumor volume variation during treatments, as follows: positive response for variations of at least −50%, neutral response for variations strictly comprised between −50% and +35%, negative responses for variations of at least +35% (33–35).

The last main data type we built in Semalytics is the ‘Annotation’ class (green area in Figure 2), which works as bridge for capturing experimental evidence. Each observation is tracked by a dedicated annotation node, and it is explicitly referred to the bioentity where the evidence has been detected. Therefore, when multiple observations occur, the same sample can be connected to several annotation nodes, pointing to different knowledge items. In Semalytics, each annotation connects one instance of an operational data to a concept in knowledge through a two-predicate-long path (Figure 3). With the predicate ‘has_annotation’, a bioentity is linked to an annotation item, which is consequently connected to a concept in the knowledge with the predicate ‘has_reference’. We introduced the intermediate annotation node in order to store meta-annotations. Indeed, we can record supplementary notes about the event of the generation of the observation (e.g. user data or analytical sessions) with other triples about the annotation node.

Furthermore, where necessary, we used data properties to link Semalytics resources to literal values in order to assert further information, such as local barcodes, genomic coordinates, gene symbols or cross references about knowledge data pointing back to identifiers in data sources (e.g. Ensembl and COSMIC).

**Table 1:** The starting topology of an experimental tree is described with the predicate ‘generates’ connecting nodes (subjects) to their direct successors (objects). To facilitate the exploration of huge collections of complex trees, we defined the new transitive property ‘hasDescendant’ to automatically infer a secondary structure able to ease data exploration, without losing original connections.

**Subject**	**Object**	
	**:generates**	**:hasDescendant ‘**(inferred)’
Root	Tissue1 Tissue2	Tissue1 Tissue2 Bioentity1 Bioentity2 Bioentity3 Bioentity4 Bioentity5
Tissue1	Bioentity1	Bioentity1 Bioentity4
Tissue2	Bioentity2 Bioentity3	Bioentity2 Bioentity3 Bioentity5
Bioentity1	Bioentity4	Bioentity4
Bioentity3	Bioentity5	Bioentity5

This modeling technique allows us to connect laboratory trees with an actionable representation of knowledge that users can harness to explore experiments and metadata without relevant modeling issues or constraints in data schema, which are typical of RDBMSs. However, data retrieval is a critical phase due to the scattered nature of annotations of hierarchical data in experimental trees. Indeed, researchers need to perform ‘intra’*-* and ‘inter’*-*tree analyses at scale. ‘Intra’*-*tree queries require the aggregation of heterogeneous observations (e.g. gene variants and drug responses) along an experimental pathway to analyze events cooccurrence. ‘Inter’*-*tree investigations are necessary in order to summarize data of more patient-related experiments and to compare different strategies. From the computational point of view, it may mean traversing hundreds of trees full of nodes to gather sparse knowledge annotations according to researcher interests. In principle, SPARQL allows those kinds of queries. However, the summarization of annotations at query runtime does not scale. Therefore, the analysis of large collections of data is often unfeasible. To address this issue, we exploited semantic reasoning to automatically enrich our ontology with several triples that can be used as logical shortcuts. In particular, we declared a new transitive property ‘hasDescendant’ as super-property of the legacy resource ‘generates’. This required only the following two triples:

:generates rdfs:subPropertyOf:hasDescendant

:hasDescendant rfd:type owl: TransitiveProperty

Once triples are loaded in the Semalytics ontology, the reasoner uses such statements to infer new information: every couple of nodes connected with ‘generates’ is consequently connected with the predicate ‘hasDescendant’. Moreover, since this property ‘R’ is transitive, given three nodes ‘a’, ‘b’ and ‘c’, the following relation holds:

aRb ∧ bRc ⇒ aRc

Therefore, a secondary tree structure is added automatically by the reasoner, connecting any node at any level with all its offspring through predicates ‘hasDescendant’. This secondary inferred structure flattens the trees connecting downstream nodes and their annotations directly to ancestors, regardless of their depth (e.g. Table 1).

Analogously, to improve the exploration of alterations, we created the property ‘has_variant’ as super-property of all predicates connecting genes with their aberrant features, independently of the variant category. This makes the reasoner add new triples, connecting each gene to all its aberrant modifications in a more general way.

The inference processes are performed by the reasoner that materializes new persistent triples. Those entailed triples can be used at query runtime at no extra computational cost. The entailment usually takes several seconds even on huge collections of trees. It is worth observing that, since Semalytics relies on the reasoner, there is no need to write complex queries or custom triggers in order to materialize new implicit information and to keep it up-to-date, as it is instead necessary in the relational model or in data models relying on plain graphs without semantics. The reasoner automatically keeps inferred statements aligned to explicit triples also after subsequent variations of data (e.g. data addition and deletion). In this way, Semalytics allows users to track, explore and summarize immediately scattered data in experimental trees, even on large collections. Without those entailed data, queries for exploring trees may take more than 30 minutes or may even become infeasible.

### Linked data integration

Semalytics aims to harness an augmented knowledge representation in order to enrich local data and improve its analytical capabilities. Even if an increasing amount of knowledge is constantly released in literature, it is locked up in documents written in natural language, or it is released in scattered datasets with different data models and barely interconnected. Currently, there is a plethora of IT-based tools for exploring biological information (7), but most of them are still largely relying on naïve metadata or natural text processing, making information hard to query and to leverage at scale. The current challenge is turning data in machine-understandable information (11, 12). The semantic framework of Semalytics addresses this issue, offering a built-in way for enriching knowledge based on the Linked Data paradigm (36). Indeed, RDF-based datasets released as Linked Data can be easily integrated with Semalytics.

Since the first introduction of Semantic Web fundamentals by Sir Tim Berners-Lee, many linked datasets related to life sciences have been released (https://lod-cloud.net), exploiting a first-class publication process for data interoperability and linkage (https://5stardata.info/en/). Although the semantic framework is a stable model, an effective connection of datasets for creating a web of biological features in translational research, as well as in many other fields, is a still critical point (37). It turned out that even integrating semantic datasets still requires a considerable effort. Copying and merging Linked Data in local triplestores is not as straightforward as it might seem at the first sight. Indeed, different designs in knowledge representation require a huge manual curation for both merging and maintenance of Linked Data. Alternatively, datasets can be combined at queries runtime with federated queries (37). With this pattern, queries are distributed over different SPARQL endpoints (i.e. linked datasets interfaces), and external information is reached hitting remote endpoints at queries runtime, with no need to maintain a local copy of remote data. Even if this approach avoids heavy datasets updates, it still needs mapping rules between local information and all the datasets exposed through remote endpoints. Moreover, running federated queries over many endpoints becomes soon infeasible because of their limited availability/up-time or poor performance of distributed queries (38, 39).

To overcome such shortcomings, we connected Semalytics to Wikidata (http://www.wikidata.org), the crowdsourced project of Wikimedia Foundation (15) based on a Linked Data model (40). A very active community, its collaborative philosophy, a CC0*-‘*based’ licensing policy, as well as a central and a highly available SPARQL endpoint make Wikidata an ideal context for centralizing integration and maintenance of biological data (41). To date, many knowledge sources are available in Wikidata, resembling a unique and interconnected living picture (42). Recent curation initiatives are focusing on the use of Wikidata as central hub for linked life sciences information, integrating and synchronizing biological data, such as human genes and variants, pathways, proteins, diseases, chemical compounds and drugs, as well as related scientific bibliography (43, 44). This makes possible building applications based on this biological data ecosystem (45). The majority of imported items in Wikidata retain also original identifiers pointing back to initial sources (e.g. Ensembl and COSMIC), as well as further metadata about each statement such as the information about the original reference. Interestingly, if there are contradictory evidence about a fact, Wikidata can store also statements about controversies (e.g. a variant can predict both a positive and a negative response to the same drug). This feature reflects the real nature of the scientific knowledge, which sometimes is debated and it is not as clear-cut as one may argue.

There is a variety of different practical approaches for linking Wikidata to other knowledge graphs (46). We connected Semalytics knowledge to Wikidata through the OWL Web Ontology Language (47). In particular, the platform relies on local knowledge for preliminary data annotation, but then local information is extended through pointers to Wikidata. We decided to map local triples with the remote ones exploiting the predicate ‘owl:sameAs’. With this property, we can define that two resources, even if identified by different URIs, are actually representing the same entity. For example, stating local triples as follows:

:n679698:symbol ‘PTEN’;

:ac ‘ENSG00000171862’;

rdf:type:Gene;

owl:sameAs wd:Q14878377.

We define ‘:n679698’ as our local representation of the gene *PTEN* with a pointer to its Ensembl identifier. Then, we assert that it is the identical concept of the resource with the URI ‘wd:Q14878377’, which is the Wikidata representation of the same gene. The resource ‘wd:Q14878377’ is also present in the remote dataset but with more details that we did not store locally. Additionally, the remote version of the resource is interconnected to an enriched network of improved and updated information that can be directly linked to local experimental data for analytical tasks.

**Figure 4:**
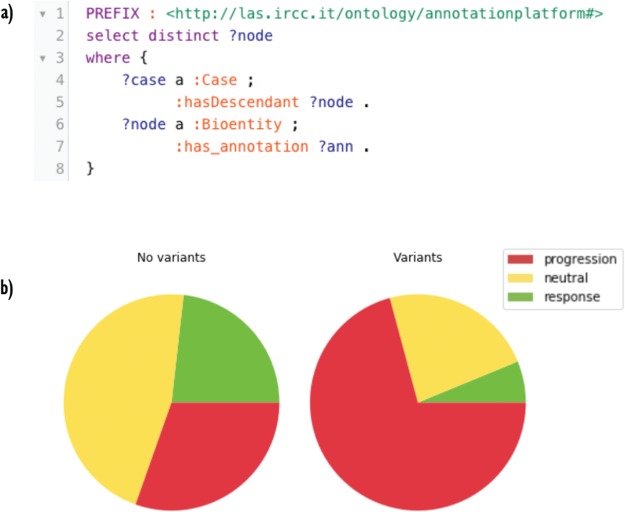
(**A**) A SPARQL query for retrieving annotated nodes. In the very first part, a ‘PREFIX’ is declared as a shorthand for local URIs. The pattern described in the ‘WHERE’ clause asks GraphDB to get all the annotated nodes. In line 2, the projection clause selects distinct nodes. (**B**) Response fractions in trees with no variants in the genes panel (on the left) and with one or more variants (on the right). Cases harboring variants in the panel are less sensitive to cetuximab.

Following this pattern, we mapped more than 24 000 human genes covering the human coding genome through their Ensembl identifiers. Moreover, matching COSMIC identifiers, we mapped about 80 variants coming from the CIViC database (48), a community-driven collection about clinical interpretation of variants in cancer, oriented to precision medicine and currently integrated in Wikidata.

Thanks to those new data identities, Semalytics allows users to (ii) federate local data with an external knowledge base in order to gain augmented insights about data interpretation.

## Querying data with Semalytics

In this section, we present a functional proof-of-concept in order to exemplify the capabilities of Semalytics in querying data and to simulate the usage of our tool in translational cancer research. In particular, we exploit the platform to analyze correlations between DNA aberrations in colorectal cancer cases and their response to drugs.

We harness Semalytics for the analysis of a dataset that we have generated for the evaluation of drugs efficacy in metastatic colorectal cancer (33) and that we had previously examined with *ad hoc* data procedures. Those data have been produced through hierarchical experiments using patient-derived samples implanted in mice (49) for administering different drugs and for propagating bioentities downstream in order to compare longitudinal genomic data. Specifically, here we investigate DNA variant types ‘sequence_alteration’ and ‘feature_amplification’ (i.e. a subclass of ‘copy_number_variation’) related to a panel of four genes (*BRAF*, *EGFR*, *HER2/ERBB2* and *KRAS*), which are known to play relevant roles in the response to a drug named cetuximab. This drug is a monoclonal antibody against the Epidermal Growth Factor Receptor that is clinically approved for treatment of several cancer types, including metastatic colorectal cancer (33, 50).

**Figure 5:**
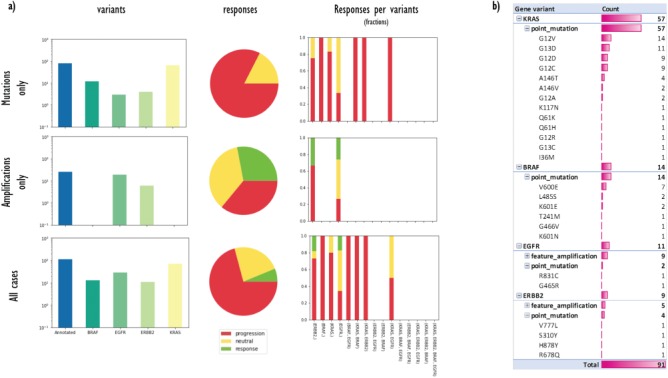
(**a**) A data matrix obtained by querying annotations scattered along experimental trees with Semalytics. Each chart in the matrix represents the combination of criteria in the relative row and column. Regarding matrix rows, the top one shows cases with mutations in the genes panel (*BRAF*, *EGFR*, *HER2*/*ERBB2* and *KRAS*) without amplifications; the middle row exhibits cases harboring amplifications in the panel without mutations; the bottom one shows cases with mutations or amplifications. Variants distributions per each gene (log-10 scale), response rates and response fractions per variants are illustrated in columns. In particular, in the third column, we correlate genomic landscapes with drug responses. Each colored bar of charts in the third column represents the response fraction of cases with aberrations detected in genes on x-axes. (**b**) Variants occurrences in non-responder cases grouped by gene, then by variant type, finally by variant instance (available only for mutations).

For this proof-of-concept, we loaded in Semalytics the LAS biobank of the Istituto di Candiolo that includes the aforementioned annotated dataset. We dumped LAS data in RDF format, then we serialized them into a Turtle file. To do this, we performed a custom preprocessing of LAS data depending on the specific data representation of the source. We turned original data in triples describing the hierarchy of the experiments and relative observations, according to the data schema of Semalytics. Then, we loaded the RDFized information into the platform database with the REST API available in GraphDB (51), thus binding the initial dataset to the Semalytics ontology and triggering inference. In this investigation, we removed annotations that are not related to the exploration scope (e.g. annotations about other gene variants) but we retained all the available operational data (i.e. about 10 000 hierarchical trees), as well as all the local knowledge representation, in order to make Semalytics deal with a large dataset. In this test, data are managed in a unique GraphDB repository with about 14 million asserted triples and more than 2 million inferred axioms. The platform runs on an HW/SW with the following features: Intel Core i7-6700 CPU at 3.40GHz, 16 GB of memory, SSD, Ubuntu 16.04, GraphDB 8.7 (as triplestore), Python 3.7.0 for handling queries and result sets. An interactive computational narrative with further details about this proof-of-concept is provided in a Jupyter notebook (52), available in the Docker-based demo bundled with this work.

### Semantic exploration of local data

First, we used Semalytics to analyze and correlate scattered annotations about genetic alterations and responses of tumors to cetuximab to test the trees exploration features of Semalytics. In this example, we focus on root nodes (i.e. starting cases) and we use the SPARQL endpoint of Semalytics to query our data (e.g. Figure 4a). Running several SPARQL queries, basic insights about the datasets can be discovered almost instantly. For example, we realized there are about 4000 bioentities with notes about genes variants in the panel or responses to the cetuximab. Moreover, 354 trees are annotated with one or more variants, while 238 have notes about drug responses. The presence of one or more variants of genes in the panel seems affecting the response to the cetuximab. Indeed, about 30% of cases with no variants has negative responses to that drug, but this fraction raises up to 70% in cases with variants in the panel (Figure 4b). Semalytics allows users to rapidly retrieve the set of the cases annotated with both genes and variants notes, which is composed by 113 trees and that represents the investigation set of the following examples.

With Semalytics, we can drill in this investigation set to mine more insights. An example of this kind of analyses is shown in Figure 5, where we matched gene variants against drug responses in different ways. For example, we analyzed the distribution of general variants per each gene, exploring the whole set, the subset ‘Amplifications only’ of cases harboring amplifications (i.e. ‘feature_amplification’) but no mutations, or the subset ‘Mutations only’ harboring mutations (i.e. ‘sequence_alteration’) but no amplifications. We discovered that in the subset ‘Amplifications only’, just two genes are amplified (i.e. *EGFR and ERBB2*) and the most amplified one is *EGFR*. Instead, the most frequently mutated gene in the subset ‘Mutations only’ is *KRAS.* From the responses point of view, the three response types are almost uniformly distributed in the subset ‘Amplifications only’, instead, the drug performs way worse in the subset ‘Mutations only’.

Besides, we used Semalytics to correlate genomic landscapes with drug responses and we calculated response fractions, filtering cases per detected aberrations for the whole set and for subsets ‘Amplifications only’ and ‘Mutations only’ (third column of Figure 5a). In particular, we computed response fractions of cases with variants in single genes and with variants cooccurring in more than one gene. It turned out that are no cases with variants cooccurring in all the genes in the panel or with variants cooccurring in three genes. There are only several cases harboring alterations in two genes at the same time and they resulted not sensitive to the drug. The cetuximab seems effective only on several cases harboring *EGFR* or *ERBB2* amplifications.

Finally, we used Semalytics to explore the genomic variants detected in all trees annotated with negative responses. In particular, genomic alterations of non-responder cases are sliced and diced to present distributions of altered genes, alteration types per gene (mutations or amplifications) and mutations detected per gene. As shown in Figure 5b, those cases are characterized by mutations in *KRAS* and *BRAF*, as well as several amplifications in *EGFR* and *ERBB2*. In particular, about 85% of investigated variants are point mutations while the others are amplifications. Variants occur mainly in *KRAS* (63%), while remaining alterations are almost equally distributed across the other three genes.

### Querying data with linked knowledge

Semalytics harnesses its architecture also to connect local information to external knowledge maintained remotely (i.e. Wikidata), in order to extend the analytical viewpoint. The investigation set we explored in the section above is saved in the local database but can be analyzed also through the knowledge stored remotely to uncover response predictions to different drugs. We ran the following queries on November 2018. Wikidata information is continuously maintained and updated; therefore slightly different results may be returned in the future.

We used the platform to retrieve chemical compounds, stored in Wikidata, which physically interact with products encoded by genes included in the investigation panel. The rationale behind this is that when the genes of the panel are altered, their protein products are aberrantly activated and profoundly affect the behavior of cancer cells. Therefore, a drug targeting such altered protein products can potentially confer a clinical benefit interfering with the tumor biology. In this case, the platform selects remote knowledge about drug–gene interactions and retrieves local cases harboring mutations in genes for which specific drugs have been reported. This analytical pipeline can be exploited to generate new hypotheses. Indeed, the repositioning of existing drugs in new therapeutic contexts can be inferred thanks to the integration of genomic data and available gene–drug interaction annotations. In turn, such hypotheses can then be challenged experimentally by going back to the biological samples. In the specific setting of this investigation set, approximately 20 different drug–gene options are returned from Wikidata. Such data analyses are usually managed by Semalytics with one federated query that hits the Wikidata endpoint.

Furthermore, we can use Semalytics to explore remote information about explicit variants evidence. The rationale behind this new analysis pattern is that biomarkers in experimental data (e.g. the gene mutation *BRAF V600E*) can predict positive or negative responses to drugs, even if those evidence come from therapies for different cancer types. Leveraging the platform, we can prioritize hypotheses about other drugs that can be validated. Using Semalytics for this investigation set, we discovered several dozen positive and negative response predictions about local observations. Moreover, information about the publication record describing such evidence is available for further details. For example, we found that samples in our local investigation set harboring the mutation *BRAF V600E* can be potentially treated with Dabrafenib/Trametinib combination therapy (https://www.wikidata.org/wiki/Q38160427). Indeed, this therapeutic option has a good performance in patients affected by melanomas characterized by that alteration, as stated in the literature returned through the platform (53–55). Also in this case, we got such data with a unique federated query in Semalytics.

**Figure 6:**
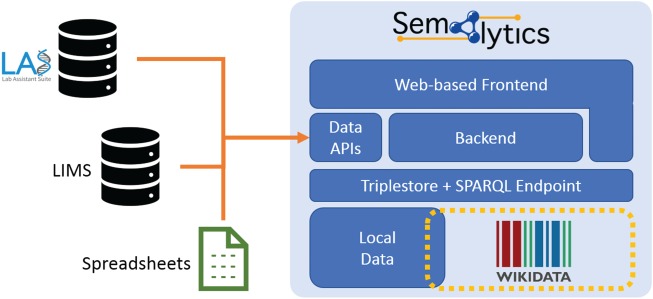
Deployment of Semalytics as a Web-based platform. Local data are mapped to Wikidata creating an annotation system with extended knowledge. Those two data spaces can be federated with SPARQL. The backend and the Web frontend help users interact with data, even if they are not familiar with SPARQL query language. Finally, data Application Programming Interfaces manage information ingestion from external sources (e.g. LIMSs or spreadsheets), as well as the exporting of results of local data processing. Those two data spaces can be federated with SPARQL. The backend and the Web frontend help users interact with data, even if they are not.

## Discussion and future work

In this work, we presented the data framework of Semalytics, the core of an IT platform that combines (i) an efficient exploration of scattered and heterogeneous data along hierarchical experimental trees with (ii) the connection to structured knowledge in Wikidata. This platform allows the storage and the analysis of large collections of hierarchical data, which are typical of several research settings in translational pharmacogenomics. We based the architecture of this tool on Semantic Web and Linked Data technologies in order to provide a structured representation of operational data and related observations. The local data framework is expanded and completed with logical reasoning and external knowledge in Wikidata. Finally, we deployed a proof-of-concept analysis to demonstrate the effectiveness and the efficiency of the platform capabilities.

The semantic data schema we introduced in Semalytics tackles main issues of RDBMSs in managing this data scenario. Indeed, this data schema can be easily modified or updated, and it deals efficiently with graph and tree structures. The connection with Wikidata provides a high-quality hub for biological knowledge that avoids the need for local knowledge curation, which is usually effort-consuming and prone to human errors. Wikidata is a living knowledge base where a very active community keeps data up-to-date and continuously loads new information. The effort of this data curation is distributed among a large group of users and collected in a central interconnected repository. Moreover, very large datasets, including those related to biological knowledge, are loaded and maintained through programmatic bulk operations. Interestingly, the more Wikidata is used, the better is the quality of its data. For example, during the building and the usage of Semalytics, we found some minor data issues that we corrected or that we reported to Wikidata curators and so they promptly fixed them. Moreover, this connection hits just one external SPARQL endpoint, thus minimizing technical issues such as network latency and federation performance.

As we mentioned, Wikidata is a remote collaborative platform that anyone can edit. This approach may have several drawbacks on data control and availability for federated analyses. Indeed, data vandalisms, errors, as well as unavailability of the remote endpoint, can temporarily compromise the results of federated queries. During our tests, we noticed that data damages are extremely rare and that the downtime of Wikidata endpoint is negligible (56). Besides, operational data can be loaded locally and connected to local Wikidata references, even if the remote endpoint is temporarily off-line. Eventually, local dumps of Wikidata can be stored and deployed locally, for example with a custom installation of Wikibase (i.e. the software collection used by Wikidata (57)), and then connected to Semalytics. These versions can be used as a knowledge extension backup during eventual Wikidata downtimes, or also to reproduce static results that are not affected by the evolution of the remote knowledge base.

Semalytics is a support system for translational investigators for generating and prioritizing *in silico* hypotheses that can be subsequently tested in a laboratory. For example, we imagine several main use cases in drugs assessment. As we showed in the proof-of-concept, Semalytics can be used to summarize drugs performance against genomic landscapes in longitudinal experiments. Moreover, researchers can exploit the platform to automatically connect their experimental results to remote evidence in order to select drugs that could be potentially effective given the observed genomic landscapes. Semalytics could be used also for alternative analytical patterns. For instance, the platform can be exploited to verify consensus about evidence. Indeed, in local experimental data, some genomic landscapes could be annotated with positive responses to a drug but, in the remote knowledge, there could be a negative response prediction to such a drug for such genomic features, or vice versa. Those cases could be object of supplementary laboratory experiments for discovering different biological dynamics. Moreover, the platform can also be extended for the investigation of other biological features relevant to translational research, even for non-hierarchical data. Altogether, this platform deploys an IT infrastructure to harness machines not only to store data but also to track and connect a machine-understandable representation of the information, in order to save effort for data preprocessing that researchers can spend for an improved data investigation.

As future developments, we planned to enrich Semalytics with graphical interfaces for a user-friendly data exploration that does not require one to master SPARQL. Improvements may also involve the integration of several Application Programming Interfaces in the backend devoted to data ingestion from external sources and to data export. With these enhancements, Semalytics can be distributed as a full-fledged Web-based platform with a Docker-based installation, bundling a free-licensed version of a triplestore (Figure 6).

## Notes

**Database URL (data core demo):** https://github.com/lasircc/semalytics-demo

## References

[1] Greaves M. and Maley C.C. (2012) Clonal evolution in cancer. Nature, 481, 306. 22258609 10.1038/nature10762 PMC3367003

[2] Collins F.S. and Varmus H. (2015) A new initiative on precision medicine. N. Engl. J. Med., 372, 793–795. 25635347 10.1056/NEJMp1500523 PMC5101938

[3] Byrne A.T., Alférez D.G., Amant F. et al. (2017) Interrogating open issues in cancer precision medicine with patient-derived xenografts. Nat. Rev. Cancer, 17, 254. 28104906 10.1038/nrc.2016.140

[4] Tentler J.J., Tan A.C., Weekes C.D. et al. (2012) Patient-derived tumour xenografts as models for oncology drug development. Nat. Rev. Clin. Oncol., 9, 338. 22508028 10.1038/nrclinonc.2012.61 PMC3928688

[5] Beerenwinkel N., Greenman C.D. and Lagergren J. (2016) Computational cancer biology: an evolutionary perspective. PLoS Comput. Biol., 12, e1004717. 10.1371/journal.pcbi.1004717 PMC4742235 26845763

[6] Wetterstrand K.A. DNA Sequencing Costs: Data from the NHGRI Genome Sequencing Program (GSP). Available at: www.genome.gov/sequencingcostsdata. Accessed: 2018-11-02.

[7] Lu Z. (2011) PubMed and beyond: a survey of web tools for searching biomedical literature. Database, 2011, baq036. 21245076 10.1093/database/baq036 PMC3025693

[8] Chen Y., Lin Y., Yuan X. et al. (2016) LIMS and clinical data management In: Translational Biomedical Informatics. Springer, Singapore, pp. 225–239. 10.1007/978-981-10-1503-8_9 27807749

[9] Cerami E., Gao J., Dogrusoz U. et al. (2012) The cBio cancer genomics portal: an open platform for exploring multidimensional cancer genomics data. Cancer Discovery, 2012 May; 2:401–4. 22588877 10.1158/2159-8290.CD-12-0095 PMC3956037

[10] Conte N., Mason J.C., Halmagyi C. et al. (2018) PDX finder: a portal for patient-derived tumor xenograft model discovery. Nucleic Acids Res., 47, D1073–D1079. 10.1093/nar/gky984 PMC6323912 30535239

[11] Howe D., Costanzo M., Fey P. et al. (2008) Big data: the future of biocuration. Nature, 455, 47. 18769432 10.1038/455047a PMC2819144

[12] Goble C. and Stevens R. (2008) State of the nation in data integration for bioinformatics. J. Biomed. Inform., 41, 687–693. 18358788 10.1016/j.jbi.2008.01.008

[13] Berners-Lee T., Hendler J. and Lassila O. (2001) The semantic web. Sci. Am., 284, 34–43.11396337

[14] Shadbolt N., Berners-Lee T. and Hall W. (2006) The semantic web revisited. IEEE Intell. Syst., 21, 96–101.

[15] Vrandečić D. and Krötzsch M. (2014) Wikidata: a free collaborative knowledgebase. Commun. ACM, 57, 78–85.

[16] Han J., Haihong E., Le G. and Du J. (2011, October) Survey on NoSQL database In: 2011 6th International Conference on Pervasive Computing and Applications. IEEE, Port Elizabeth, South Africa, pp. 363–366.

[17] Vicknair C., Macias M., Zhao Z. et al. (2010, April) A comparison of a graph database and a relational database: a data provenance perspective In: Proceedings of the 48th Annual Southeast Regional Conference. ACM, p. 42.

[18] Miller J. J. (2013, March) Graph database applications and concepts with Neo4j. In: Proceedings of the Southern Association for Information Systems Conference, Atlanta, GA, USA, **2324**.

[19] Băzăr C. and Iosif C.S. (2014) The transition from RDBMS to NoSQL. A comparative analysis of three popular non-relational solutions: Cassandra, MongoDB and Couchbase. Database Syst. J., 5, 49–59.

[20] Baralis E., Bertotti A., Fiori A. et al. (2012) LAS: a software platform to support oncological data management. J. Med. Syst., 36, 81–90. 10.1007/s10916-012-9891-6 23117791

[21] Fiori A., Grand A., Alberto P. et al. (2018) A case study of a laboratory information system developed at the institute for cancer research at Candiolo In: Biomedical Engineering: Concepts, Methodologies, Tools, and Applications. IGI Global, Penn., USA, pp. 505–531.

[22] Fiori A., Grand A., Geda E. et al. (2018) LAS: a bio-clinical integrated laboratory information system for translational data management In: Emerging Developments and Practices in Oncology. IGI Global, Penn., USA, pp. 56–93.

[23] Grand A., Geda E., Mignone A. et al. (2019) One tool to find them all: a case of data integration and querying in a distributed LIMS platform. Database, 2019, baz004. 10.1093/database/baz004 PMC6352757 30698777

[24] Zerbino D.R., Achuthan P., Akanni W. et al. (2017) Ensembl 2018. Nucleic Acids Res., 46, D754–D761. 10.1093/nar/gkx1098 PMC5753206 29155950

[25] Forbes S.A., Beare D., Gunasekaran P. et al. (2014) COSMIC: exploring the world's knowledge of somatic mutations in human cancer. Nucleic Acids Res., 43, D805–D811. 25355519 10.1093/nar/gku1075 PMC4383913

[26] Lassila O. and Swick R.R. (1999) Resource Description Framework (RDF) Model and Syntax Specification.

[27] Hitzler P., Krotzsch M. and Rudolph S. (2009) Foundations of Semantic Web Technologies. Chapman and Hall/CRC, FL., USA.

[28] Berners-Lee T. and Hendler J. (2001) Publishing on the semantic web. Nature, 410, 1023. 11323639 10.1038/35074206

[29] Ter Horst H.J. (2005) Completeness, decidability and complexity of entailment for RDF schema and a semantic extension involving the OWL vocabulary. Web Semant., 3, 79–115.

[30] Beckett D. (2008) Turtle-Terse RDF Triple Language. http://www.ilrt.bris.ac.uk/discovery/2004/01/turtle/. Accessed date: 02 November 2018.

[31] Hanahan D. and Weinberg R.A. (2000) The hallmarks of cancer. Cell, 100, 57–70. 10647931 10.1016/s0092-8674(00)81683-9

[32] Hanahan D. and Weinberg R.A. (2011) Hallmarks of cancer: the next generation. Cell, 144, 646–674. 21376230 10.1016/j.cell.2011.02.013

[33] Bertotti A., Migliardi G., Galimi F. et al. (2011) A molecularly annotated platform of patient-derived xenografts ('xenopatients') identifies HER2 as an effective therapeutic target in cetuximab-resistant colorectal cancer. Cancer Discov., CD–11. 10.1158/2159-8290.CD-11-0109 22586653

[34] Bertotti A., Papp E., Jones S. et al. (2015) The genomic landscape of response to EGFR blockade in colorectal cancer. Nature, 526, 263. 26416732 10.1038/nature14969 PMC4878148

[35] Zanella E.R., Galimi F., Sassi F. et al. (2015) IGF2 is an actionable target that identifies a distinct subpopulation of colorectal cancer patients with marginal response to anti-EGFR therapies. Sci. Transl. Med., 7, 272ra12–272ra12. 10.1126/scitranslmed.3010445 25632036

[36] Bizer C., Heath T. and Berners-Lee T. (2011) Linked data: the story so far In: Semantic Services, Interoperability and Web Applications: Emerging Concepts. IGI Global, Penn., USA, pp. 205–227.

[37] Prud’hommeaux E. and Buil-Aranda C. (2013) SPARQL 1.1 Federated Query. W3C Recommendation, 21, 113.

[38] Buil-Aranda C., Hogan A., Umbrich J. et al. (2013, October) SPARQL web-querying infrastructure: ready for action? In: International Semantic Web Conference. Springer, Berlin, Heidelberg, pp. 277–293.

[39] Rakhmawati N.A. and Hausenblas M. (2012, September) On the impact of data distribution in federated sparql queries In: 2012 IEEE Sixth International Conference on Semantic Computing. IEEE, Palermo, Italy, pp. 255–260.

[40] Hernández D., Hogan A. and Krötzsch M. (2015) Reifying RDF: What Works Well with Wikidata? SSWS@ ISWC, 1457, Workshop at the International Semantic Web Conference in Bethlehem, Penn., USA, pp. 32–47.

[41] Mitraka E., Waagmeester A., Burgstaller-Muehlbacher S. et al. (2015) Wikidata: a platform for data integration and dissemination for the life sciences and beyond, 10.1101/031971.

[42] Wikidata Statistics https://www.wikidata.org/wiki/Wikidata:Statistics(03 April 2019, date last accessed).

[43] Good B.M., Burgstaller-Muehlbacher S., Mitraka E. et al. (2016, August) Opportunities and challenges presented by Wikidata in the context of biocuration In: From International Conference on Biological Ontologies 2016, Oregon State University, Corvallis, OR, USA.

[44] Burgstaller-Muehlbacher S., Waagmeester A., Mitraka E. et al. (2016) Wikidata as a semantic framework for the gene wiki initiative. Database, 2016, baw015. 26989148 10.1093/database/baw015 PMC4795929

[45] Putman T.E., Lelong S., Burgstaller-Muehlbacher S. et al. (2017) WikiGenomes: an open web application for community consumption and curation of gene annotation data in Wikidata. Database, 2017, bax025. 10.1093/database/bax025 PMC5467579 28365742

[46] Waagmeester A., Willighagen E.L., Queralt-Rosinach N. et al. (2016) Linking Wikidata to the rest of the semantic web. SWAT4(HC)LS (Semantic Web Applications and Tools for Healthcare and Life Sciences), Amsterdam, The Netherlands.

[47] McGuinness D.L. and Van Harmelen F. (2004) OWL Web Ontology Language Overview. W3C Recommendation, 10, 2004.

[48] Griffith M., Spies N.C., Krysiak K. et al. (2017) CIViC is a community knowledgebase for expert crowdsourcing the clinical interpretation of variants in cancer. Nat. Genet., 49, 170. 28138153 10.1038/ng.3774 PMC5367263

[49] Hidalgo M., Amant F., Biankin A.V. et al. (2014) Patient-derived xenograft models: an emerging platform for translational cancer research. Cancer Discov., 4, 998–1013. 25185190 10.1158/2159-8290.CD-14-0001 PMC4167608

[50] Jonker D.J., O'callaghan C.J., Karapetis C.S. et al. (2007) Cetuximab for the treatment of colorectal cancer. N. Engl. J. Med., 357, 2040–2048. 18003960 10.1056/NEJMoa071834

[51] The RDF4J Server REST API http://docs.rdf4j.org/rest-api/#_repository_statements (03 April 2019, date last accessed).

[52] Kluyver T., Ragan-Kelley B., Pérez F. et al. (2016, May) Jupyter Notebooks-A Publishing Format for Reproducible Computational Workflows. In: The International Conference on Electronic Publishing (ELPUB) - Göttingen, Germany, pp. 87–90.

[53] Menzies A.M. and Long G.V. (2014) Dabrafenib and trametinib, alone and in combination for BRAF-mutant metastatic melanoma. Clin. Cancer Res., clincanres–2054. 10.1158/1078-0432.CCR-13-2054 24583796

[54] Long G.V., Hauschild A., Santinami M. et al. (2017) Adjuvant dabrafenib plus trametinib in stage III BRAF-mutated melanoma. N. Engl. J. Med., 377, 1813–1823. 28891408 10.1056/NEJMoa1708539

[55] Long G.V., Stroyakovskiy D., Gogas H. et al. (2014) Combined BRAF and MEK inhibition versus BRAF inhibition alone in melanoma. N. Engl. J. Med., 371, 1877–1888. 25265492 10.1056/NEJMoa1406037

[56] Wikidata Query Service (WDQS) Endpoint usage: https://discovery.wmflabs.org/wdqs/#endpoint_usage (03 April 2019, date last accessed).

[57] Wikibase (docker version): https://github.com/wmde/wikibase-docker (03 April , date last accessed).

